# Targeting aerosol delivery to regions of nasal-associated lymphoid tissue (NALT) in three dimensional models of human intranasal airways using the BiVax intranasal atomizer

**DOI:** 10.3389/fddev.2024.1456538

**Published:** 2024-11-11

**Authors:** Beth L. Laube, Jana Kesavan, Gonçalo Farias, Nektaria Karavas, Mathilde Blondel, Julie Suman

**Affiliations:** ^1^ Independent Researcher, Annapolis, MD, United States; ^2^ Independent Researcher, Catonsville, MD, United States; ^3^ Aptar Pharma, Le Vaudreuil, France; ^4^ Aptar Pharma, Congers, NY, United States

**Keywords:** 3D models of human intranasal airways, targeted aerosol delivery, nasal-associated lymphoid tissue (NALT), model age, model head position, insertion angle, insertion distance

## Abstract

**Introduction:**

Well-organized nasal-associated lymphoid tissue (NALT) has been identified in the pharyngeal and tubal tonsils of both adults and children, and diffuse NALT has been identified in the superior, middle and inferior turbinate regions of children. However, it is not clear how to target these NALT sites with aerosolized vaccines. We explored whether head position and/or angle and distance of device insertion could be used to target fluorescein aerosol to NALT sites in three-dimensional printed models of the intranasal airways of an 18- and a 5-year-old (yo).

**Methods:**

Three head positions (upright [Up], tilted back 45° [45] and supine [Su]), two angles of insertion (30° and 45°) and two distances of insertion (6 mm and 9 mm) were tested. Fluorescein aerosol was generated by an Aptar Pharma BiVax 200 µL intranasal atomizer. Percent fluorescein deposition was quantified in the anterior nose, the upper horizontal third of the model (superior turbinate region), middle third (middle turbinate), lower third (inferior turbinate and nasopharynx combined) and exit filter.

**Results:**

Mean percent deposition in both models was <0.5% in the upper third and on the exit filter for all test conditions. A multivariate analysis showed that deposition in either model was unaffected by the angles of insertion and distances of insertion. However, middle third deposition was significantly higher in the 5-yo than in the 18-yo (*p* = 0.01) and anterior nose deposition was higher in the 18-yo than in the 5-yo (*p* < 0.01). When data from both models were combined, middle third deposition was highest in the supine position with Up < 45 < Su (*p* < 0.01) and lower third deposition was highest in the upright position with Up > 45 > Su (*p* = 0.03).

**Discussion:**

These results suggest that, in individuals with similar nasal airway dimensions as our models: 1) supine and upright head positions might be used to target delivery of aerosolized vaccines generated by the BiVax intranasal atomizer to NALT sites in the middle turbinate and the inferior turbinate and nasopharynx combined, respectively; 2) delivery to the middle turbinate may be higher in children ≤5-yo; and 3) deposition in the anterior nose may be higher in adults, for all head positions. *In vivo* tests are needed to confirm these findings.

## Introduction

Many respiratory infections are caused by viral and bacterial pathogens. These include group A streptococci, *Streptococcus* pneumoniae, *Haemophilus* influenzae, *Mycoplasma* pneumoniae, *Mycobacterium tuberculosis*, influenza virus, parainfluenza viruses, and respiratory syncytial virus) (Czerkinsky and Holmgren, 2010) and, more recently, SARS-CoV-2. Since the nasal cavity is a site of first contact for these pathogens, intranasal vaccination could be an efficacious route for early prevention of infection and transmission.

Vaccination via the intranasal route is very appealing. It has been shown to induce immunity in the respiratory system as well as in the digestive and reproductive systems and to increase systemic immune responses (Casteleyn et al., 2010). In addition, the nose is easily accessible for vaccination by both the caregiver and for self-administration. It also eliminates the pain associated with vaccination by injection as well as the possibility of the spread of pathogens by contaminated needles, important to mass vaccination campaigns. Furthermore, vaccines presented to the nasal mucosa are less vulnerable to dilution and enzymatic attack, compared to presentation to the gut epithelium via oral vaccination (Baumann, 2008).

Two drugs have been licensed for intranasal administration to stimulate intranasal immunity and prevent influenza. These are FluMist/Fluenz and Nasovac™. Other drugs are being developed for nasal delivery to prevent respiratory syncytial virus (Blue Lake Biotechnology, 2022) and COVID-19 (Chavda et al., 2021).

Successful immunization requires the presence of lymphoid tissue. In the nasal cavity, this tissue is called NALT, which is an abbreviation used to describe nasal-associated lymphoid tissue (Sabirov and Metzger, 2008), nasal-cavity associated lymphoid tissue (Casteleyn et al., 2010), and nasopharynx-associated lymphoid tissue (Brandtzaeg and Pabst, 2004). In adults and children, well-organized NALT is found in the pharyngeal and tubal tonsils, located in the nasopharynx (Koornstra et al., 1991; Kuper et al., 1992; Kuper et al., 2003; Boyaka et al., 2000). In addition, diffuse NALT has been identified in the superior, middle and inferior nasal turbinates of young children (Debertin et al., 2003).

Successful vaccination via the intranasal route likely also requires targeted delivery of aerosolized vaccine to NALT sites and there are many factors that could affect such delivery. (Djupesland et al., 2014; Williams and Suman, 2022; Le Guellec et al., 2021). A number of investigators have shown that three-dimensional (3D) models, based on computed tomography (CT), or magnetic resonance imaging (MRI), scans of human intranasal airways, can be used in preclinical development of intranasal aerosol delivery devices to quantify intranasal deposition under various test conditions (Warnken et al., 2018; Pires et al., 2009; Dugernier et al., 2019; Möller et al., 2008; Durand et al., 2011; Leclerc et al., 2015; Maniscalco et al., 2006; Sato et al., 1981; Möller et al., 2015; Castile et al., 2013). Models are created from the scans using stereolithography and 3D printing technologies.

We previously investigated the effect of head position on regional aerosol distribution in 3D printed models based on the intranasal airways of an 18-year-old and a 5-year-old. Aerosol was generated by a MAD™ atomizer (Teleflex, Morrisville, NC). Results from that study showed that aerosol deposition in the inferior turbinate and the nasopharynx region combined (inferior turbinate/nasopharynx), was higher when the 18-year-old model head was upright and tilted backwards at 45°, compared to the model head supine and tilted backwards at 45° (Kesavan J. S. et al., 2023). For the 5-year-old model, deposition in the inferior turbinate/nasopharynx region was higher when the model head was upright and tilted backwards at 45°, or was supine, compared to supine with the head tilted backwards at 45° (Kesavan J. S. et al., 2023). This suggested that head position might be used to target NALT sites for intranasal vaccination. However, we administered the aerosol with the atomizer pointed upward towards the ear and the middle of the internal nasal valve area of the models, as suggested in the MAD™ package insert, and others have shown that more horizontal angles (i.e., 30° and 45°) improve deposition to the inferior and middle turbinate regions in 3D models, compared to steeper angles (i.e., 60° and 75°) (Warnken et al., 2018). In addition, Akash et al. used computational fluid dynamic models to investigate the effect of particle size on targeting aerosol deposition to NALT sites. They reported that aerosols with particles smaller than those generated by the MAD™ atomizer are needed to target deposition to the nasopharyngeal region (Akash et al., 2023).

In the present study, we further explored head position as well as angle of insertion and distance of insertion, as factors that could target aerosol delivery to sites associated with NALT in 3D printed models of intranasal airways. We also tested for the effect of age by including an adult and child model and we used the BiVax intranasal atomizer, which generated particles similar in size to those recommended by Akash et al. The goal was to identify aerosol- and patient-related factors that might be used to target aerosol delivery of vaccines to sites associated with NALT regions.

## Materials and methods

### Model construction and components

A 3D model based on the head and intranasal airways of a 5-year-old African American male was constructed as described previously (Laube et al., 2015). A 3D model based on the head and intranasal airways of an 18-year-old male of unknown ethnicity was similarly constructed. The 18- year-old model was produced from CT scans of an 18-year-old individual in the supine position and with the same eligibility criteria as described for the 5-year-old model (Laube et al., 2015).

Each model included a soft flexible face with a nose with patent nostrils, interior nasal vestibule and nasal valve area (anterior nose), and the main nasal airway, which was divided into three horizontal pieces (upper third, middle third and lower third). The 5-year-old model also included a closed mouth. Previously, we have shown that the intranasal anatomy of both models is similar to published anatomy for humans of similar ages (Kesavan J. S. et al., 2023). We have also demonstrated that deposition in the 18-year-old model is similar to *in vivo* deposition for adults (Kesavan J. et al., 2023).

The upper third of the main nasal airway includes the superior turbinate region and olfactory cleft, as well as the frontal, ethmoid and sphenoid sinuses, as present on the CT scans. The middle third includes the middle turbinate region, as well as the maxillary sinuses, as present on the CT scans. The lower third includes the inferior turbinate region and the nasopharynx combined (inferior turbinate/nasopharynx).

Photos of the two models have been published previously (Laube et al., 2015). The anterior nose and three horizontal pieces are detachable, so they can be studied separately. A schematic diagram showing the sectioning of the models for regional deposition analysis has been published previously (Laube et al., 2015).

### Experimental procedure

A Design of Experiment (DOE) was utilized to test six conditions (n = 3) for each model. The order for testing each condition was determined from the DOE. The DOE was an optimal experimental design with an algorithm that aimed to minimize the prediction variance. A total of 36 tests was conducted in the two 3D models combined. During the 36 tests, each model head was positioned upright (Up), tilted back 45° (45), or supine (Su). These head positions were selected because there is no consensus as to the best head position for administering nasal aerosols. Some package inserts for nasal sprays indicate patients should lean forward slightly during administration (Asthma and Allergy Network, 2023). Others suggest they should tilt their head backward (Thomas et al., 2013; Milk et al., 2021), or inhale while in an upright position (Cleveland Clinic Health Essentials, 2022).

Two insertion angles (30° and 45°) were selected for these experiments, based on previous findings that these two angles enhance deposition in the middle and inferior turbinate regions of 3D printed models, compared to 60° and 75° angles (Warnken et al., 2018). The two insertion distances (6mm and 9 mm) were selected because some instructions state the patient should insert the tip of the nasal spray ¼ to ½ inch (6.35 mm–12.7mm, respectively) into their nose (Asthma and Allergy Network, 2023).

**FIGURE 1:**
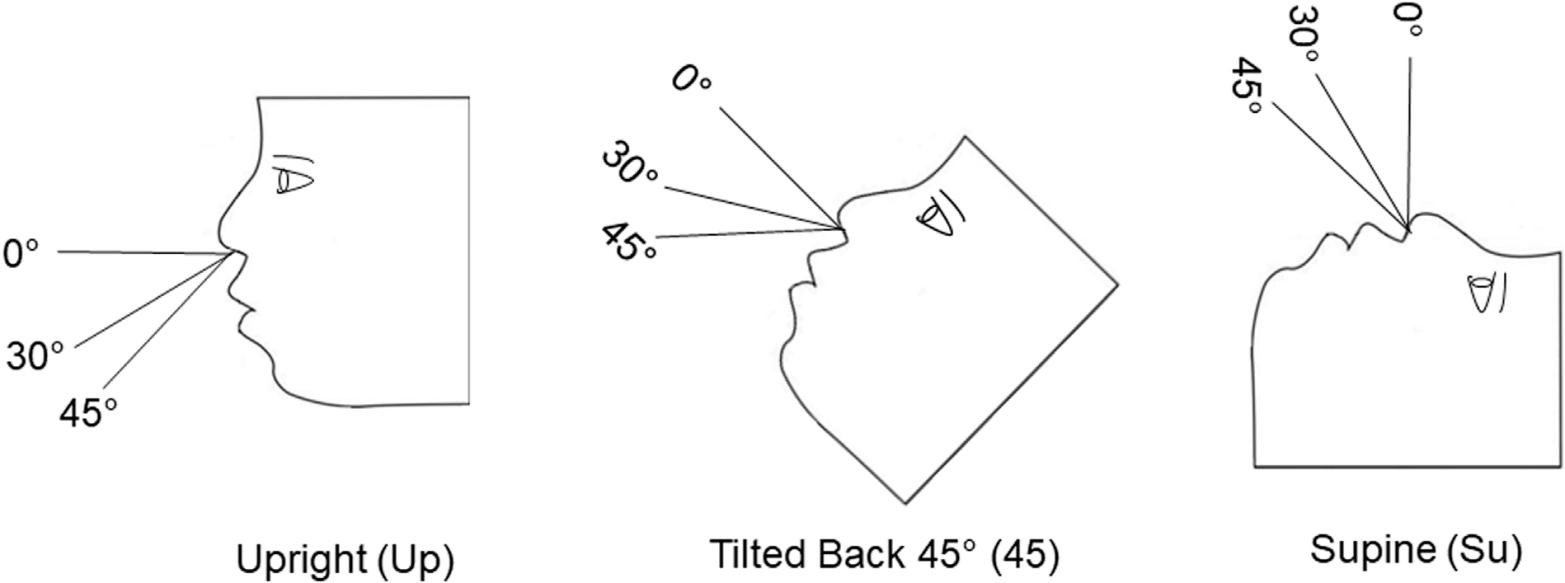
Cartoon showing the three model positions [upright (Up), tilted back at 45° (45) and supine (Su)] and the two angles of insertion (30° and 45°) that were tested.

Figure 1 shows the three head positions and the two insertion angles that were used in these experiments. For the 18-year-old model, the six conditions included: Up-30°-6mm, Up-45°-9mm, 45–30°-9 mm, 45–45°-6 mm, Su-30°-6 mm, Su-45°-9 mm. For the 5-year-old model, the six test conditions included: Up-30°-6 mm, Up-45°-9 mm, 45–30°-6 mm, 45–45°-9 mm, Su-30°-9 mm, and Su-45°-6 mm.

### Aerosol generation and administration

**FIGURE 2:**
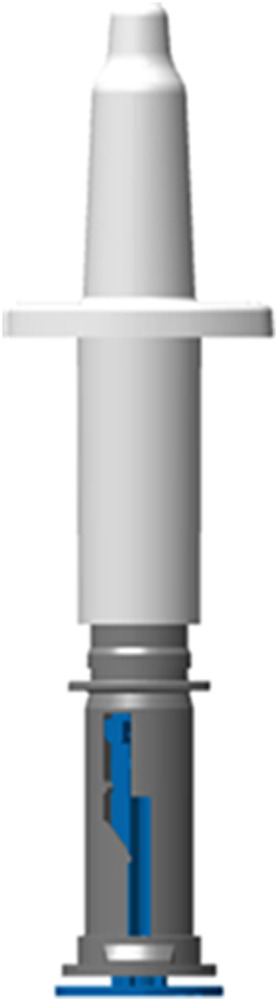
Photo of the BiVax 200 µL intranasal atomizer.

Aerosol was generated from a solution of distilled water admixed with 0.005 mg/mL sodium fluorescein (fluorescent solution) and was administered into each model nostril using an Aptar Pharma BiVax 200 µL intranasal atomizer that is shown in Figure 2. For each experiment, an unused BiVax was loaded with 200 µL of the fluorescent solution as follows. The white nozzle was removed, the syringe needle was inserted into the fluorescent solution, and 200 µL was drawn into the syringe (i.e., 100 µL for administration into each nostril). Then, the white nozzle was placed back over the needle and pushed in fully until a “click” was audible.

**FIGURE 3:**
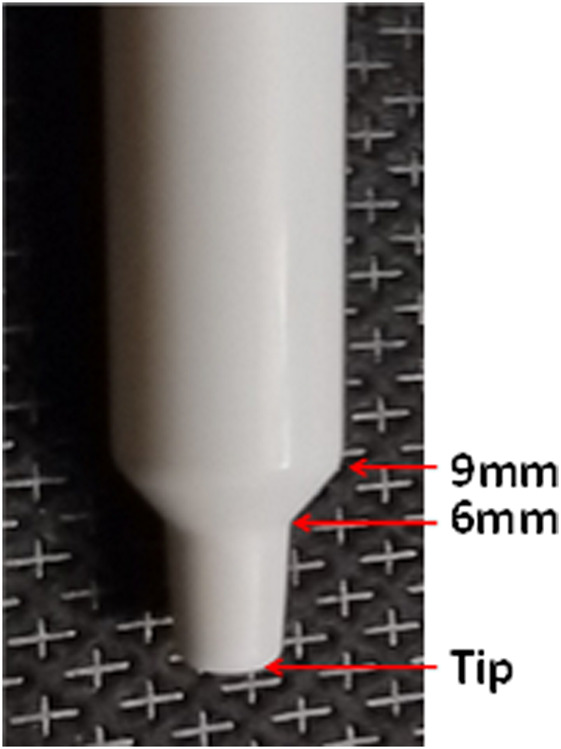
Photo of the BiVax intranasal atomizer showing the insertion distances of 6 and 9 mm marked on the white nozzle.

Model heads were positioned as shown in Figure 1, according to the DOE. The BiVax delivery system was inserted 4° from the center of each nostril and away from the septum. The objective was to center the sprayer nozzle in the nasal vestibule to improve deposition in the posterior nasal cavity. The insertion distance and insertion angle were specified by the DOE. Figure 3 shows the two distances of insertion (6mm and 9 mm) on the BiVax™ nozzle. The plunger was depressed and 100 µL of the fluorescent solution was aerosolized into the nostril. Within 60–90 s, the piston of the atomizer was turned ¼ turn counter-clockwise and the same aerosolization procedure was repeated for the left nostril. A Type A/E filter (Thomas Scientific, Swedesboro, NJ) was attached to the base of the model to capture any aerosol that bypassed the intranasal airways (i.e., exit filter).

To seal the model sections, we lightly coated the interior borders of each section with petroleum jelly (Equate, distributed by Walmart). We also surrounded each model with Velcro straps. These steps resulted in tight connections between sections. The interior anatomy of each model section was not coated.

To reduce possible movement of liquid between sections due to gravitational forces, we kept the time between spray administrations and the disassembly of the model sections to a minimum (i.e., 2–3 min total) for all three head positions and test conditions. We also placed the model in the supine position immediately upon removal from the jig and disassembly of the sections to further limit liquid movement between sections due to gravity.

Aerosol was delivered under static breathing conditions (i.e., there was no airflow through the model during aerosolization). We did not incorporate airflow into the experimental design because there is no consensus as to whether there should be airflow during intranasal aerosol delivery. Some package inserts instruct patients to inhale gently, or not at all (Asthma and Allergy Network, 2023).

### Control of head position, insertion angles and distances during aerosol administration

Two wooden jigs were constructed to control head position, insertion angles and distances during aerosol administration. One jig was constructed for the 18-year-old model and another was constructed for the 5-year-old model. Each jig allowed for administration of the aerosol in the Up, 45, or Su head position, with insertion angles 30° or 45°, relative to 0° as shown in Figure 1, and at an insertion distance of 6mm, or 9mm, as shown in Figure 3.

**FIGURE 4:**
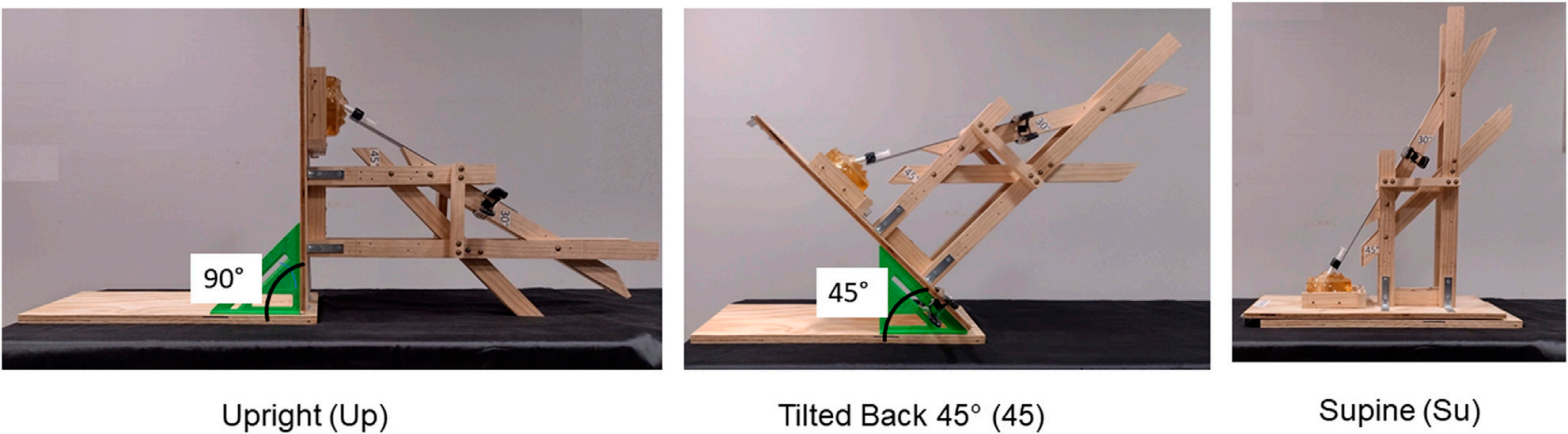
Photos of the jig that controlled head position and angle of insertion of the BiVax 200 µL intranasal atomizer in the 5-year-old model. The angle of insertion is 30° for all three positions. Panel A shows the model in the upright position. Panel B shows the model with the head tilted backwards at 45°. Panel C shows the model in the supine position.

Jigs consisted of two wooden pieces connected by a hinge that allowed for the head to move independently from Su to 45 to Up. Two additional wooden pieces (guides) were attached to the jig at 30° and 45°, respectively, for each nostril. A detachable metal strip lay flat on each guide during aerosol administration. The BiVax atomizer was attached to the metal strip, which was adjusted so that the atomizer could be inserted 6mm, or 9mm, into each nostril. A clamp was used to hold the metal strip with the atomizer in place for each experiment. Figure 4 shows the jig for the 5-year-old model with the BiVax atomizer and model in the three head positions during administration at the 30° insertion angle.

### Quantification of droplet size distribution and spray plume angle

We quantified droplet size and spray plume angle for an aerosol that was generated by the BiVax 200 µL atomizer with deionized water (no fluorescein). Since sodium fluorescein is fully soluble in water (Jampol and Cunha-V, 1984), the viscosity of the water solution and the fluorescent solution should be similar. Therefore, we assumed that the addition of fluorescein should have no impact on spray performance as measured for the water aerosol, and droplet size and spray plume angle should be the same for the two solutions.

Both droplet size distribution (DSD) and spray pattern tests were conducted using automated actuation with a Vereo^®^ Actuator NSx (Proveris Scientific; Hudson, MA) at a human representative velocity of 50 mm/s until a force of 6 kg was achieved at a distance that ensured a representative spray was captured with an acceptable discriminatory ability. Table 1 shows the actuation parameters.

**TABLE 1:** Parameters associated with automated actuation for the Bivax 200 µL intranasal atomizer.

Automated actuation parameters	Value
Velocity (mm/s)	50
Hold Time (ms)	300
Actuation Mode	Force
Contact Force (Kg)	0.3
End of Stroke Force (Kg)	6

DSD was determined via laser diffraction using a Malvern Spraytec™ (Malvern Instruments, Worcestershire, UK). Two doses were tested at 4 cm from the laser from each of five BiVax 200 µL atomizers. Average DSD was expressed as the volume median diameter [Dv(50)]. Parameters associated with the quantification of Dv(50) are found in Table 2.

**TABLE 2:** Parameters associated with quantification of Dv(50) for the Bivax 200 µL intranasal atomizer.

Malvern acquisition method	Value
Acquisition frequency (Hz)	500
Acquisition duration (ms)	150
Trigger: transmission (%)	93
Analysis distance (cm)	4
Distance nozzle/lens (cm)	9

Spray plume angle was determined using a SprayView (Proveris Scientific; Hudson, MA), with the BiVax atomizer positioned 5 cm from the laser. Two doses were tested from each of five BiVax 200 µL atomizers. Spray plume angle was expressed in degrees. Parameters associated with the quantification of spray plume angle are found in Table 3.

**TABLE 3:** Parameters associated with quantification of spray plume angle for the BiVax 200 µL intranasal atomizer.

Parameters	Value
Camera Height (cm)	32
Camera Distance (cm)	8
Frame Rate (Hz)	100
Frames to acquire	100
Iris Setting	2
Threshold	6
Spray Duration	Dose 1: start time 200 ms - end time 500 ms Dose 2: start time 200 ms - end time 400 ms
Pattern Distance (cm)	5

### Sample recovery

After aerosol administration, the model pieces were separated and placed into individual plastic bags. Liquid that dripped from any model piece was collected on a Type A/E filter, that was placed in the bag with its corresponding model piece. The filter attached at the base of the model was removed and placed in a 50 mL centrifuge tube.

Twenty mL of pH-corrected distilled water was placed in each bag with a model piece and in the centrifuge tube with the filter. Bags were shaken by hand every 5 min, during a 15-min period, to remove deposited fluorescein. Tubes were continuously shaken on a digital orbital shaker (ONiLAB, City of Industry, CA) for 15 min.

### Fluorescent measurements

Approximately 3 mL of recovery solution was withdrawn from each bag containing a model piece and from the tube containing the filter. Each sample was placed in a culture tube and measured for fluorescence using a fluorometer (Sequoia-Turner Model 450 Fluorometer, Barnstead/Thermolyne, Dubuque, IA), according to methods published previously (Kesavan et al., 2001).

### Cleaning model pieces

After each test, all model pieces were washed with water containing Dawn soap (Proctor and Gamble, Cincinnati, OH) and then rinsed with tap water to remove the fluorescein. Complete removal of fluorescein was determined prior to the next test by placing washed model pieces in clean bags, adding 20 mL of recovery solution, shaking the bags every 5 min during a 15- minute period and measuring the fluorescence in a sample from that solution. If the readings were not zero, the washing procedure was repeated. Three repetitions, or less, of this washing procedure was sufficient to obtain zero readings. Models were air dried before the next test.

### Calculation of aerosol deposition

Deposition in the anterior nose, upper, middle and lower horizontal thirds, and on the exit-filter was determined from their fluorometer measurements and was expressed as a percentage of the sum of the fluorescent measurements of all the model pieces and the exit-filter. Percent deposition in the upper, middle and lower thirds is presented separately and as a combination of all three pieces combined (total turbinates).

### Data analysis

Percent deposition is presented as mean ± 1 standard deviation (SD). A multivariate analysis was performed to determine the influence of each variable (model age, model position, insertion angle and insertion depth) on deposition distribution using SOS Stat software (version 3.5.0.6, Doussard, France). The impact of model age was determined by combining the data for all six tests for the 5-year-old model vs a combination of all six tests for the 18-year-old model. The impact of model position was determined by combining data from both models for the lower and middle thirds. Data for the filter and upper third of the two models were not included in the multivariate analysis because deposition in these sections was <0.5%. Unpaired t-tests were conducted to evaluate the influence of each coefficient linked to each variable (i.e., model age, model position, angle of insertion and insertion depth) and interaction (i.e., model age/model position, model age/angle of insertion, model age/insertion depth, model position/angle of insertion) in the multivariate model. Certain interactions (angle/insertion depth, model position/insertion depth and quadratic terms) were excluded, as these have been widely explored in the literature (Kesavan J. S. et al., 2023; Le Guellec et al., 2021; Williams and Suman, 2022). *p* < 0.05 indicated statistical significance for each coefficient.

## Results

### Droplet size distribution

The average Dv(50) ± standard deviation was 36 ± 1.0 µm.

### Spray plume angle

The average spray plume angle ±standard deviation was 68.9° ± 2.1°.

### Timing between sprays and model disassembly

On average, there was approximately 60–90 s between the time of the first and second sprays and approximately 90 s between the second spray, removal of the model from the jig, placement of the model in the supine position and disassembly into sections.

### Deposition

**FIGURE 5:**
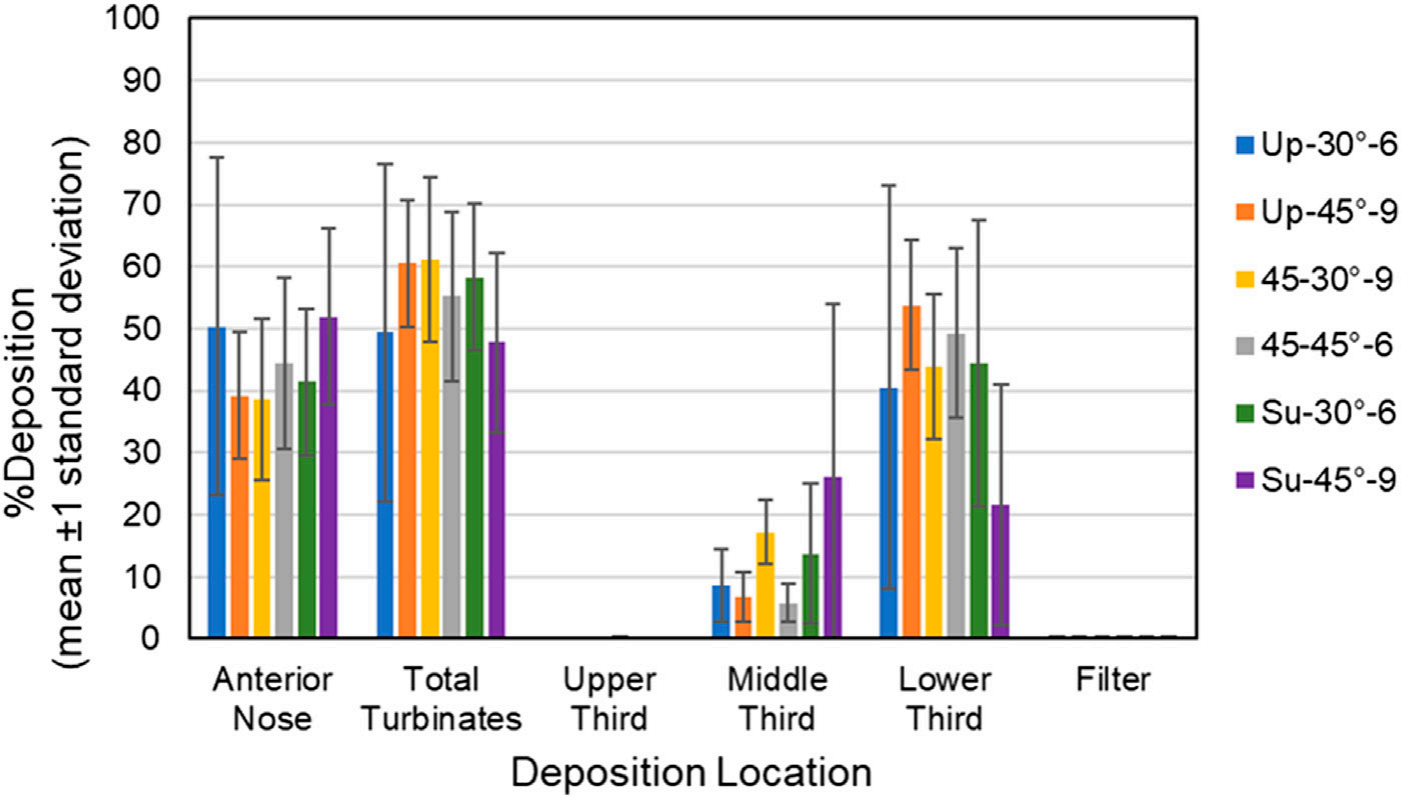
Bars showing mean percent deposition ±1 standard deviation in each region of the 18- year-old model for all six test conditions. Color codes provide the parameters for each test condition (i.e., model position, insertion angle, insertion distance).

**FIGURE 6:**
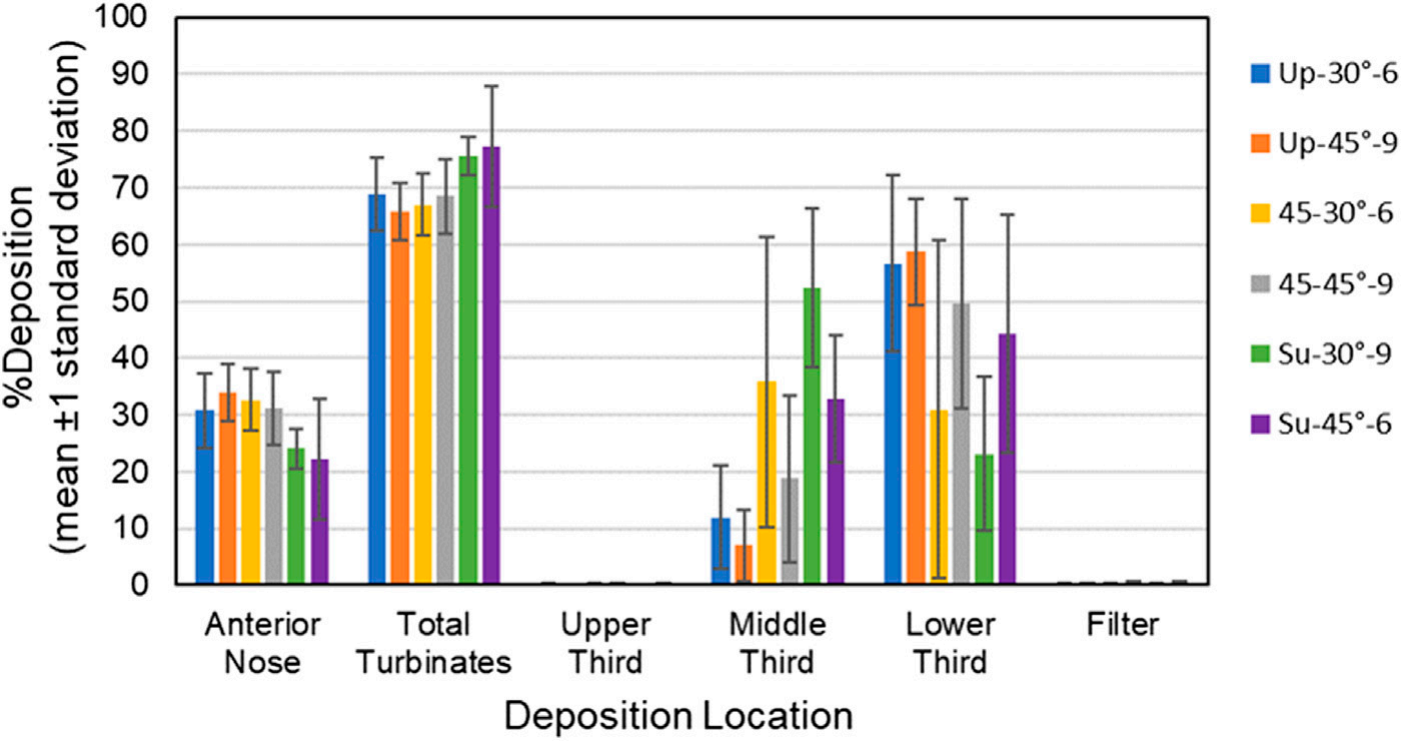
Bars showing mean percent deposition ±1 standard deviation in each region of the 5-year-old model for all six test conditions. Color codes provide the parameters for each test condition (i.e., model position, insertion angle, insertion distance).

Mean percent aerosol deposition ±1 standard deviation for each of the six test conditions for the anterior nose, total turbinates, upper third, middle third, lower third and filter for the 18-year- old model and 5-year-old model is summarized in Tables 4–7, respectively. Mean percent deposition ±1 standard deviation at each deposition location for the 18-year-old model and 5-year-old model is shown graphically in Figures 5, 6, respectively.

**TABLE 4:** Mean (M) percent aerosol deposition ±1 standard deviation (SD) for test conditions 1, 2 and 3 for the six regions in the 18-year old-model.

Test condition	1		2		3	
Position	Upright		Upright		45	
Angle	30°		45°		45°	
Insertion Distance	6 mm		9 mm		6 mm	

	Deposition Percent					
Model Region	M	SD	M	SD	M	SD
Anterior Nose	50.38	27.26	39.22	10.11	44.43	13.77
Total Turbinates	49.33	27.21	60.50	10.12	55.23	13.70
Upper Third	0.10	0.11	0.11	0.09	0.15	0.11
Middle Third	8.72	5.84	6.68	3.97	5.81	2.95
Lower Third	40.50	32.55	53.72	10.49	49.27	13.71
Filter	0.29	0.05	0.28	0.08	0.34	0.15

**TABLE 5:** Mean (M) percent aerosol deposition ±1 standard deviation (SD) for test conditions 4, 5 and 6 for six regions in the 18-year-old model.

Test condition	4		5		6	
Position	45		Supine		Supine	
Angle	30°		30°		45°	
Insertion Distance	9 mm		6 mm		9 mm	

	Deposition Percent					
Model Region	M	SD	M	SD	M	SD
Anterior Nose	38.55	13.10	41.39	11.80	51.95	14.29
Total Turbinates	61.13	13.17	58.34	11.90	47.73	14.34
Upper Third	0.08	0.06	0.09	0.08	0.06	0.03
Middle Third	17.23	5.24	13.76	11.29	26.15	27.90
Lower Third	43.83	11.60	44.49	23.08	21.52	19.37
Filter	0.31	0.07	0.28	0.10	0.32	0.05

**TABLE 6:** Mean (M) percent aerosol deposition ±1 standard deviation (SD) for test conditions 1, 2 and 3 for the six regions in the 5-year-old model.

Test condition	1		2		3	
Position	Upright		Upright		45	
Angle	30°		45°		30°	
Insertion Distance	6 mm		9 mm		6 mm	

	Deposition Percent					
Model Region	M	SD	M	SD	M	SD
Anterior Nose	30.78	6.52	33.92	5.03	32.63	5.46
Total Turbinates	68.86	6.49	65.78	4.96	67.02	5.50
Upper Third	0.19	0.16	0.04	0.02	0.26	0.12
Middle Third	11.96	9.10	7.05	6.31	35.82	25.55
Lower Third	56.70	15.48	58.69	9.41	30.94	29.81
Filter	0.36	0.16	0.30	0.08	0.35	0.14

**TABLE 7:** Mean (M) percent aerosol deposition ±1 standard deviation (SD) test conditions 4, 5 and 6 for six regions in the 5-year-old model.

Test condition	4		5		6	
Position	45		Supine		Supine	
Angle	45°		45°		30°	
Insertion Distance	9 mm		6 mm		9 mm	

	Deposition Percent					
Model Region	M	SD	M	SD	M	SD
Anterior Nose	31.02	6.42	22.24	10.68	24.04	3.40
Total Turbinates	68.53	6.51	77.32	10.65	75.67	3.35
Upper Third	0.20	0.17	0.30	0.07	0.11	0.08
Middle Third	18.77	14.62	32.72	11.16	52.42	13.95
Lower Third	49.56	18.41	44.31	21.05	23.14	13.53
Filter	0.45	0.10	0.44	0.18	0.29	0.13

**Anterior Nose:** For the 18-year-old model, mean percent deposition ranged between 38.55% and 51.95%, with maximum deposition at Su-45°-9 mm and minimum at 45–30°-9 mm. For the 5-year- old model, mean percent deposition ranged between 22.24% and 33.92%, with maximum deposition at Up-45°-9 mm and minimum at Su-45°-6 mm.

**Total Turbinates:** For the 18-year-old model, mean percent deposition ranged between 47.73% and 61.13%, with maximum deposition at 45–30°-9 mm and minimum deposition at Su-45°-9mm. For the 5-year-old model, mean percent deposition ranged between 65.78% and 77.32%, with maximum deposition at Su-45°-6 mm and minimum deposition at Up-45°-9 mm.

**Upper Third:** For the 18-year-old model, mean percent deposition in the upper third was ≤0.2% for all test conditions. For the 5-year-old model, mean percent deposition in the upper third was ≤0.3% for all test conditions.

**Middle Third:** For the 18-year-old model, mean percent deposition ranged between 5.81% and 26.15%, with maximum deposition at Su-45°-9 mm and minimum deposition at 45–45°-6 mm. For the 5-year-old model, mean percent deposition ranged between 7.05% and 52.42%, with maximum deposition at Su-30°-9 mm and minimum deposition at Up-45°-9 mm.

**Lower Third:** For the 18-year-old model, mean percent deposition ranged between 21.52% and 53.72%, with maximum deposition at Up-45°-9 mm and minimum deposition at Su-45°-9 mm. For the 5-year-old model, mean percent deposition ranged between 23.14% and 58.69%, with maximum deposition at Up-45°-9 mm and minimum deposition at Su-30°-9 mm.

**Filter:** For both models, mean percent deposition on the filter was <0.5% for all test conditions.

### Dripping

On three of the 36 test runs, one drop of liquid was observed dripping from the nostril of the model following aerosol administration. All three runs were in the Up position. The drop was captured on a Type A/E filter, added to the bag with the anterior nose piece, and counted as part of the fluorescence for that piece. Dripping occurred from the 18-year-old model during the run with a 30°insertion angle and 6 mm insertion distance and during the run with a 45°insertion angle and 9 mm insertion distance. Dripping occurred from the 5-year-old model during the run with a 30°insertion angle and 6 mm insertion distance. No dripping from the interior of the assembled turbinate sections to the exterior borders of either model was observed under any test conditions. In addition, there was no observed dripping from the upper, middle, or lower third sections of the models after disassembly, under any test condition.

### Multivariate analysis

The multivariate analysis showed that deposition in sites associated with NALT was similar using 30° or 45° angles of insertion, and 6 or 9 mm insertion distances (Table 8). However, model age significantly affected anterior nose deposition, which was significantly higher in the 18-year-old, compared to the 5-year-old model (*p* < 0.01). In addition, model age (5-year-old vs 18-year-old) significantly affected middle third and total turbinate deposition, which was significantly higher in the 5-year-old, compared to the 18-year-old model (*p* = 0.01 and *p* < 0.01, respectively) (Table 8). In addition, when data from both models were combined, head position significantly impacted middle and lower third deposition. Middle third deposition was highest in the supine head position with Up < 45<Su (*p* < 0.01) and lower third deposition was highest in the upright head position with Up > 45>Su (*p* = 0.03) (Table 8).

**TABLE 8:** Results of multivariate analysis of variance. Model age (MA) (i.e., 5-year-old vs. 18-year- old) had an impact on deposition in the anterior nose (higher for 18-year-old; *p* < 0.01) and the middle third and total turbinates (higher for 5-year-old; *p* = 0.01, *p* < 0.01, respectively). Model position (MP) had an impact on deposition in the middle third (Up < 45<Su; *p* < 0.01) and lower third (Up > 45>Su; *p* = 0.03). Regional deposition was no different using 30° or 45° angles of insertion, or 6 mm or 9 mm insertion distances (all *p* > 0.05). The last four columns provide interaction effects for model age and position, model age and angle of insertion, model age and insertion distance and model position and angle of insertion. Interaction effects were not significant.

Model region	p Value MA	p Value MP	P Value Angle	p Value Distance	p Value MA/MP	p Value MA/Angle	p Value MA/Distance	p Value MP/Angle
Anterior Nose	<0.01	0.47	0.97	0.69	0.28	0.56	0.31	0.23
Middle Third	0.01	<0.01	0.06	0.18	0.07	0.20	0.90	0.64
Lower Third	0.80	0.03	0.20	0.25	0.55	0.21	0.59	0.29
Total Turbinates	<0.01	0.47	0.96	0.69	0.28	0.56	0.31	0.23

## Discussion

This study explored the use of head position, insertion angle and insertion distance to target the delivery of an aerosol generated by the BiVax 200 µL intranasal atomizer to sites associated with NALT in 3D printed models based on the intranasal airways of an 18-year-old and a 5-year- old. A multivariate analysis of variance showed that aerosol delivery to NALT sites was significantly affected by head position in both models. Middle third deposition was highest when models were in the supine head position. Lower third deposition was highest when models were in the upright head position. These results suggest that, in individuals with similar nasal airway dimensions as our models, supine and upright head positions might be used to target delivery of aerosolized vaccines generated by the BiVax intranasal atomizer to NALT sites in the middle turbinate and inferior turbinate region-nasopharynx, respectively.

The finding that deposition could be targeted to either the middle turbinate region, or the inferior turbinate region/nasopharynx, by administering the aerosol in different head positions may be significant, since it is not clear if the nasopharynx is the only target site for immunization. The important role nasopharynx-associated lymphoid tissue plays in human immunity is evidenced in a recent longitudinal study that suggests that children who undergo removal of their tonsils and adenoids before the age of 9 are at risk for increased respiratory infections and diseases in later life (Byars et al., 2018). However, other research suggests that diffuse NALT may also play a role in immunization. Casteleyn has quantified and characterized nasal-associated lymphoid tissue in rabbits and found that the volume, type and location of the tissue is similar to humans. In that study, he identified lymphoid tissue similar to human tonsils in the caudal region of the rabbits as well as diffuse NALT tissue located mainly in the mucosa of the middle turbinate region (Casteleyn et al., 2010). Debertin reported similar findings of diffuse NALT in young children. They examined histological specimens taken from children who had died in the first 2 years of life and found no organized NALT in the nasal tissue blocks. Instead, they found evidence of diffuse NALT in the superior, middle and inferior turbinate regions (Debertin et al., 2003). Although diffuse NALT has not yet been identified in adults, it may be an important an immunological site in children. If this is the case, results from this study indicate that alterations in head position could be a way to target both NALT sites for intranasal vaccination, and this could be particularly important for successful, future intranasal vaccination in children.

The multivariate analysis also showed that deposition in the middle third of the 5-year-old model was higher than in the 18-year-old. One explanation for this finding could be the effect of airway geometry associated with the age of the models on the mechanisms of aerosol deposition for aerosol particles generated by the BiVax atomizer (i.e., impaction and sedimentation). Total surface area and volume was 50% less in the 5-year-old model, compared to the 18-year-old model (Kesavan J. S. et al., 2023), and the corresponding differences in airway geometry would likely have altered the amount of particle deposition by impaction and sedimentation mechanisms in the two models.

We also found that significantly more aerosol deposited in the anterior nose of the 18-year-old model, compared to the 5-year-old. This result could also be due to differences in airway geometry, since the average minimum cross-sectional area (MCA) for the 5-year-old model is larger (0.90 cm^2^) than for the 18-year-old model (0.57 cm^2^) (Kesavan J. S. et al., 2023). Theoretically, the larger MCA in the 5-year-old model could lead to less impaction in the anterior nose, compared to the 18 year-old model and more aerosol penetrating to the main nasal cavity.

The multivariate analysis also showed that the selected angles of insertion and distances of insertion resulted in similar deposition fractions at NALT sites. However, it is not known if angles of insertion that are more horizontal (i.e., <30°) could further improve aerosol delivery to the middle turbinate region, and/or the inferior turbinate region/nasopharynx. In addition, it might be difficult to incorporate a more horizontal angle during delivery because of patient discomfort. Similarly, studies designed to look at the effect of distances of insertion greater than 9 mm may not be useful, since such distances also would likely be uncomfortable for some patients.

All test conditions resulted in <0.5% aerosol deposition in the upper third region of both models, suggesting that these approaches and delivery device may not be useful in targeting the superior turbinates, olfactory region, or frontal, or ethmoid sinuses with aerosolized drugs. However, this also means that vaccines delivered by this atomizer and under these test conditions are not likely to pose a significant risk for uptake in the olfactory region, thereby, avoiding possible CNS complications.

All test conditions also led to <0.5% aerosol deposition on the exit filter, indicating that very little aerosol would be available for delivery to the lower airways with these approaches. However, this also could be a benefit for vaccine delivery, since exposure of the lower airways to aerosolized live-attenuated vaccine is to be avoided.

Previously, we quantified the effect of head position on aerosol deposition in the same regions of interest and in the same 18- and 5-year-old models as in the present study using a MAD atomizer (Kesavan J. S. et al., 2023). Differences in the outcome measures for the two studies make it difficult to compare these findings statistically. In the present study, we report deposition in terms of mean ± standard deviation, whereas, in the previous study, we reported deposition in terms of median (range). However, some non-statistical observations are possible and these could be tested in future studies.

In the present study, average deposition in the anterior nose in the 45 position for the two test conditions for the 18-year-old model (i.e., 44.43% and 38.55% in Tables 4, 5, respectively) was 41.5%. In the Su position, average deposition in the anterior nose for the two test conditions (i.e., 41.39% and 51.95% in Table 5) was 46.7%. For the 5-year-old model, average deposition in the anterior nose in the 45 position for the two test conditions (i.e., 32.63% and 31.02% in Tables 6, 7, respectively) was 31.8%. In the Su position, average deposition in the anterior nose for the two test conditions (i.e., 22.24% and 24.04% in Table 7) was 23.1%. In the previous study, median deposition in the anterior nose of the 18-year-old model for the same head positions was 22% and 19%, respectively, and 9% and 14%, respectively, in the 5-year-old model. Interestingly, percentages from the previous study are similar to those reported by Hosseini et al. in another study that also used the MAD device in 3D models based on the intranasal airways of a 50- and 5- year-old (Hosseini et al., 2019).

The explanation for the observed deposition differences between the two devices does not appear to involve droplet size, since the Dv(50) of aerosol generated by the MAD atomizer was much larger than for the BiVax aerosol, with 99.6 ± 27.0 µm and 36.0 ± 1.0 µm, respectively. Theoretically, the larger particles of the MAD device should have impacted to a greater extent in the anterior nose than the smaller particles generated by the BiVax device. One explanation for the observed differences could involve the angle of device insertion. The atomizer in our previous study was aimed at the anterior nasal valve area of the models, which was a steeper angle of insertion than was used in the present study and may not be suitable for targeting the NALT. This angle may have resulted in more aerosol penetrating beyond the nasal valve and less deposition in the anterior nose, whereas, the opposite may have been true with the more horizontal angles used in the present study.

Insertion depth probably did not pay a role in the observed differences in deposition, since the insertion depth of the MAD device was within the same range as that of the BiVax atomizer. The applicator tip of the MAD device was inserted approximately 3 mm into the nostril opening, compared to 6mm and 9 mm for the BiVax atomizer.

The spray plume angle for the BiVax atomizer (68.9° ± 2.1°) may also have contributed to enhanced deposition in the anterior nose. Foo et al. quantified deposition in the anterior and turbinate regions of the Guilmette model (based on MRI slices of the intranasal airways of a 50- year-old male) for aerosols with different spray plume angles at an angle of insertion of 30° and found that deposition in the anterior region was greater for aerosols with wider spray plume angles (i.e., deposition with 69° angles >50° angles) (Foo et al., 2007).

In the present study, we also noted that average deposition in the lower third in the 45 head position for the two test conditions for the 18-year-old model (i.e., 49.27% and 43.83% in Tables 4, 5, respectively) was 46.6%. For the 5-year-old model, average deposition in the lower third in the 45 head position for the two test conditions (i.e., 30.94% and 49.56% in Tables 6, 7, respectively) was 40.2%. In contrast, median percent deposition in the same models and head position, in the previous study following administration with the MAD atomizer, was 40% and 31%, respectively. More research is needed to better understand these observations and the usefulness of these speculations.

Limitations to the present study are similar to those in other studies with these 3D printed models and have been discussed in detail previously (Kesavan J. S. et al., 2023). One of the most important limitations was that there was only one 18-year-old model and only one 5-year-old model. In addition, the 5-year-old model was based on the anatomy of an African American child, whereas the 18-year-old model was based on the anatomy of an individual of unknown race. Intranasal deposition could vary in models based on children and adults whose anatomy and airway geometry differ from those in the present study due to age, sex and/or race. In addition, the lower third piece of both the adult and child model included the inferior turbinate region and nasopharynx. Thus, quantification of deposition in this model piece did not represent deposition in the nasopharynx alone. Additional sectioning of the models in future studies are needed to address this limitation. The study also utilized a low viscosity solution (i.e., water). Additional work is needed to investigate the impact of viscosity, surface tension, and other formulation variables on deposition within these models. Although it is possible that aerosolized water that deposited on the interior surfaces of the models was affected by capillary action, such that movement of fluid occurred between neighboring sections, control of this action was beyond the scope of this study.

## Conclusion

Following aerosol delivery by the Aptar BiVax intranasal atomizer, study results showed that targeted deposition to NALT regions was affected by head position. Middle third deposition was highest when models were in the supine head position. Lower third deposition was highest when models were in the upright head position. Study findings also showed that targeted deposition to NALT regions within the models was affected by model age. Deposition in the middle turbinate region was highest in the 5-year-old model and deposition in the anterior nose was highest in the 18-year-old model. In both models, regional deposition was no different with 30° or 45° angles of insertion, or with 6 mm or 9 mm insertion distances. These results suggest that, in individuals with similar nasal airway dimensions as our models: (1) supine and upright head positions might be used to target delivery of aerosolized vaccines generated by the BiVax intranasal atomizer to NALT sites in the middle turbinate and inferior turbinate-nasopharynx combined, respectively; (2) delivery to the middle turbinate may be higher in children ≤5-yo; and (3) deposition in the anterior nose may be higher in adults, for all head positions. *In vivo* tests are needed to confirm these findings.

## Data Availability

The raw data supporting the conclusions of this article will be made available by the authors, without undue reservation.
